# Microcystin‐LR Triggers Renal Tubular Ferroptosis Through Epigenetic Repression of GPX4: Implications for Environmental Nephrotoxicity

**DOI:** 10.1002/advs.202514349

**Published:** 2025-11-30

**Authors:** Shaoru Zhang, Qi Gao, Yi Peng, Huan Zhang, Qi Shen, Meihong Guo, Yuqing Gong, Lei Chu, Weidong Wu, Yanting Wen, Wangsen Cao, Yong Wang, Lihui Wang

**Affiliations:** ^1^ The People's Hospital of Danyang & Affiliated Danyang Hospital of Nantong University Danyang 212300 China; ^2^ State Key Laboratory of Analytical Chemistry for Life Science & Jiangsu Key Laboratory of Molecular Medicine Medical School Nanjing University Nanjing 210093 China; ^3^ Medical Research Center & Department of Nephrology Northern Jiangsu People's Hospital Yangzhou 225001 China; ^4^ Center for Reproductive Medicine and Obstetrics and Gynecology Nanjing Drum Tower Hospital Affiliated Hospital of Medical School Nanjing University Nanjing 210093 China; ^5^ Danyang Center for Disease Control and Prevention Danyang 212300 China

**Keywords:** DNA methylation, ferroptosis, GPX4, Microcystin‐LR, nephrotoxicity

## Abstract

Environmental toxins represent a growing public health concern. Microcystin‐LR (MC‐LR), a potent cyanobacterial toxin found in freshwater ecosystems, has been linked to multisystem toxicity. However, its impact on renal pathology ‐ particularly through regulated cell death ‐ remains poorly characterized. This study investigates the molecular basis of MC‐LR‐induced nephrotoxicity in murine models, focusing on ferroptosis and epigenetic regulation. Using both acute and chronic MC‐LR exposure paradigms, marked kidney fibrosis and ferroptosis are observed, evidenced by lipid peroxidation, mitochondrial damage, and collagen deposition. Mechanistically, MC‐LR suppressed transcription of glutathione peroxidase 4 (GPX4) in tubular epithelial cells. This downregulation is associated with promoter hypermethylation, increased expression of DNA methyltransferases DNMT1 and DNMT3a, and enhanced recruitment of the transcriptional repressor E2F4 and co‐repressor NCoR. Notably, MC‐LR directly bound DNMT1 and DNMT3a, stabilizing their protein levels by blocking proteasomal degradation. Pharmacological inhibition of DNA methyltransferases (SGI‐1027) or ferroptosis (ferrostatin‐1) significantly ameliorated renal injury. These findings uncover a previously unrecognized epigenetic mechanism by which MC‐LR drives ferroptosis and kidney damage. Targeting the DNMT‐GPX4 axis may offer therapeutic opportunities for mitigating toxin‐induced organ injury and protecting public health against environmental biohazards.

## Graphical abstract

MC‐LR stabilizes DNMT1/3a by blocking their ubiquitin‐mediated degradation, leading to *Gpx4* promoter hypermethylation and E2F4/NCoR‐associated transcriptional repression, which drives renal tubular ferroptosis in mice. Pharmacological inhibition of DNA methylation (SGI‐1027) or ferroptosis (Fer‐1) disrupts this DNMT‐GPX4 axis, thereby alleviating MC‐LR‐induced ferroptosis and nephrotoxicity.

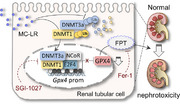

## 1 Introduction

Microcystin‐LR (MC‐LR) is one of the most toxic and well‐characterized microcystins, a group of cyclic heptapeptides produced by cyanobacteria, such as *Microcystis aeruginosa*, widely distributed in contaminated freshwater environments.^[^ ^1^, ^2^ ^]^ Human exposure to MC‐LR primarily occurs through the consumption of contaminated drinking water, agricultural products irrigated with polluted water, and aquatic food sources such as fish and shellfish.^[^ ^3^ ^]^ MC‐LR exposure has been linked to severe cytotoxicity in multiple organs, particularly the liver and kidneys, posing a serious public health risk, especially in regions affected by harmful algal blooms.^[^ ^4^, ^5^, ^6^, ^7^ ^]^ The cytotoxic effects of MC‐LR involve diverse pathological processes, such as oxidative stress, inflammatory responses, cytoskeletal disruption, and mitochondrial damage, ultimately leading to genotoxicity. These processes are closely associated with various forms of regulated cell death, including apoptosis, necrosis, pyroptosis, necroptosis, and ferroptosis.^[^ ^8^, ^9^, ^10^, ^11^ ^]^ However, the precise mechanisms underlying MC‐LR‐induced renal cell death, particularly those involving ferroptosis, remain poorly understood.

Ferroptosis, an iron‐dependent form of regulated cell death driven by lipid peroxidation, is the predominant type of cell death in renal tubules during acute kidney injury (AKI) induced by ischemia reperfusion, nephrotoxicity, or sepsis.^[^ ^12^, ^13^, ^14^ ^]^ Evidence further indicates that tubular ferroptosis initiates a maladaptive repair process that drives the progression from AKI to chronic kidney disease (CKD) and renal fibrosis.^[^ ^15^ ^]^ Although the injured epithelia possess intrinsic repair capacities and ferroptosis may permit regeneration,^[^ ^16^ ^]^ impaired repair and the failed differentiation of the regenerating epithelium lead to persistent profibrotic signaling in tubules undergoing atrophy after AKI.^[^ ^17^, ^18^ ^]^ The paracrine products released by these abnormal tubules disrupt the normal interaction between peritubular capillary endothelial cells and pericytes, thereby promoting myofibroblast transformation, proliferation, fibrosis, as well as capillary disintegration and rarefaction.^[^ ^19^ ^]^

Unlike apoptosis or necrosis, ferroptosis is not suppressed by broad‐spectrum cell death inhibitors, but can be effectively prevented by ferroptosis specific inhibitors, such as liproxstatin‐1 and ferrostatin‐1.^[^ ^20^, ^21^, ^22^ ^]^ Ferroptosis is regulated by multiple cellular pathways, involving iron, lipid, and amino acid metabolisms.^[^ ^23^ ^]^ Within this regulatory network, glutathione peroxidase 4 (GPX4) serves as a central defense by directly detoxifying lipid peroxides.^[^ ^24^ ^]^ The critical role of GPX4 in kidney tubules is underscored by findings that its inactivation triggers tubular ferroptosis and acute renal failure in mice.^[^ ^25^ ^]^ Given this pivotal function, GPX4 expression is tightly controlled. Transcriptional regulation significantly influences GPX4 abundance, and epigenetic mechanisms ‐ particularly promoter DNA methylation ‐ have emerged as an important regulatory mode.^[^ ^26^, ^27^ ^]^ Notably, the *Gpx4* promoter contains a dense CpG island, and its hypermethylation has been shown to suppress of GPX4 expression and induce ferroptosis in various cell types, including osteoblasts, nucleus pulposus cells, and lung epithelial cells.^[^ ^28^, ^29^, ^30^ ^]^ However, whether DNA methylation‐mediated epigenetic silencing of GPX4 contributes to ferroptosis in renal tubular injury remains unexplored.

DNA methylation is a fundamental epigenetic modification involving the addition of a methyl group (‐CH_3_) to cytosine residues within CpG dinucleotides,^[^ ^31^ ^]^ thereby regulating gene expression without altering the DNA sequence.^[^ ^32^ ^]^ Typically, hypermethylation of CpG in gene promoters leads to transcriptional silencing, whereas hypomethylation is associated with active transcription.^[^ ^33^, ^34^ ^]^ DNA methylation is catalyzed by DNA methyltransferases (DNMTs), a family of enzymes that includes DNMT1, which maintains methylation patterns after DNA replication, and DNMT3A and DNMT3B responsible for de novo methylation, particularly during development.^[^ ^35^, ^36^ ^]^ Conversely, Ten‐Eleven Translocation (TET) enzymes facilitate DNA demethylation by catalyzing the stepwise oxidation of 5‐methylcytosine (5mC), ultimately restoring unmethylated cytosines.^[^ ^37^ ^]^ Beyond its physiological roles, DNA methylation can be perturbed by environmental exposures. MC‐LR has been reported to exert epigenetic effects, including the modulation of DNA methylation. For example, parental exposure to MC‐LR increased methylation of the brain‐derived neurotrophic factor (BDNF) promoter in zebrafish sperm, resulting in transcriptional inhibition and transgenerational neurodevelopmental toxicity.^[^ ^38^ ^]^ However, it is unclear how it exerts the epigenetic regulatory effect.

In this study, we aimed to investigate MC‐LR nephrotoxicity with a particular focus on the epigenetic regulation of ferroptosis. Using mouse models exposed to MC‐LR through both chronic drinking water and acute intraperitoneal injection, we found that MC‐LR induces significant renal ferroptosis by directly targeting DNMT1 and DNMT3a and inhibiting their ubiquitination‐mediated degradation. This leads to hypermethylation of the *Gpx4* promoter, transcriptional suppression of GPX4 and subsequent ferroptosis, which contributes substantially to MC‐LR‐induced kidney injury. Our findings reveal a novel epigenetic mechanism underlying MC‐LR nephrotoxicity and highlight potential preventive and therapeutic strategies.

## 2 Results

### 2.1 Mice Exposed to MC‐LR Exhibit Progressive Tubular Injury, Iron Deposition and Marked Cell Death

**Figure 1:**
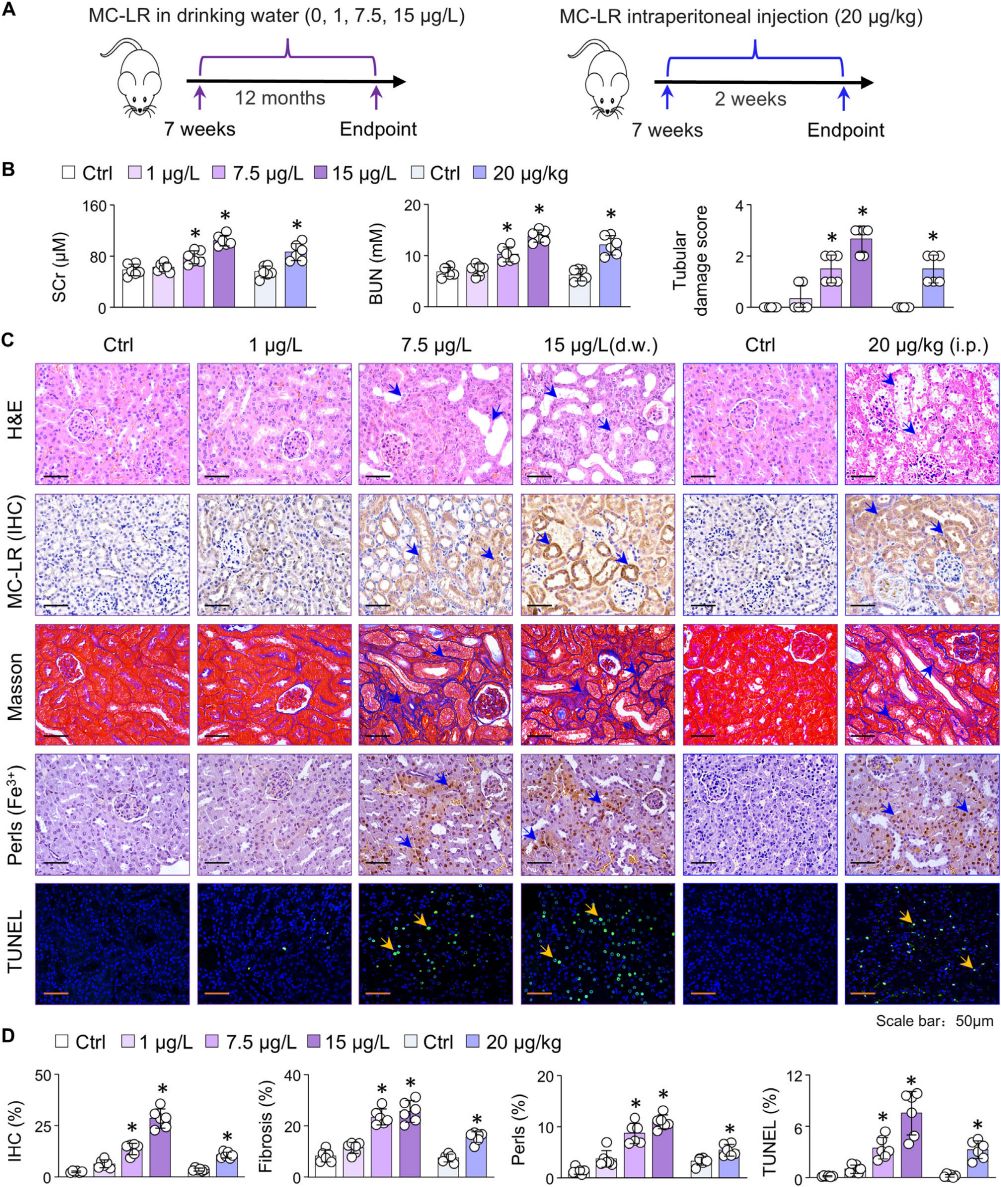
Mice exposed to MC‐LR exhibit progressive tubular injury, iron deposition, and marked cell death. A) Schematic of MC‐LR exposure mouse models. Seven‐week‐old male BALB/c mice were exposed to MC‐LR via drinking water (1, 7.5, 15 µg L^−1^) for 12 months or through daily intraperitoneal injection (20 µg kg^−1^) for 2 weeks. B) The serum levels of creatinine (SCr) and urea nitrogen (BUN) and quantification of tubular damages from control (Ctrl) and MC‐LR exposure mice. Data are presented as mean ± SD (one‐way ANOVA and Student's t‐test, *n* = 6). ^*^ *P* < 0.05 versus. Ctrl. C) Representative microscopic photographs of renal sections from Ctrl and MC‐LR exposure mice, including (H&E, MC‐LR IHC, Masson's trichrome, Perls (Fe^3+^) staining, and TUNEL). Arrows indicate tubular damage, MC‐LR accumulations, collagens, and iron depositions, and TUNEL‐positive cells. D) Quantitation of (C). Data are presented as mean ± SD (one‐way ANOVA and Student's t‐test, *n* = 6). ^*^ *P* < 0.05 versus. Ctrl.

To investigate the nephrotoxic effects of MC‐LR, we established both chronic (1, 7.5, 15 µg L^−1^ in drinking water, 12 months) and acute (20 µg kg^−1^ i.p., 2 weeks) MC‐LR exposure models in mice based on previous concentrations (**Figure** **1**A). The serum levels of creatinine and urea nitrogen and score of tubular damage were significantly elevated after MC‐LR exposure compared with the administration of the vehicle alone (Figure 1B). Hematoxylin and eosin (H&E) staining revealed significant tubular damages following MC‐LR exposure, evidenced by epithelial swelling and vacuolization, epithelial cell detachment, tubule dilation, tubule atrophy (Figure 1C). Immunohistochemistry (IHC) staining of renal tissue sections demonstrated that MC‐LR accumulated in the kidneys in a dose‐dependent manner (Figure 1C,D). Masson staining further showed increased fibrillar collagen deposition (Figure 1C,D). Prussian blue (Perls) staining revealed increased iron (Fe^3+^) deposition (Figure 1C,D). Additionally, terminal deoxynucleotidyl transferase dUTP nick‐end labeling (TUNEL) staining, a sensitive method for detecting dead cells,^[^ ^25^ ^]^ revealed substantial numbers of positive cells in the kidneys of both chronically (7.5 and 15 µg L^−1^) and acutely‐exposed mice (Figure 1C,D). Together, these results demonstrate that MC‐LR accumulates in the kidneys, leading to severe tubular damage, fibrosis‐like lesions, iron deposition, and marked cell death.

### 2.2 MC‐LR Induces Ferroptotic Alterations in Mouse Kidneys

**Figure 2:**
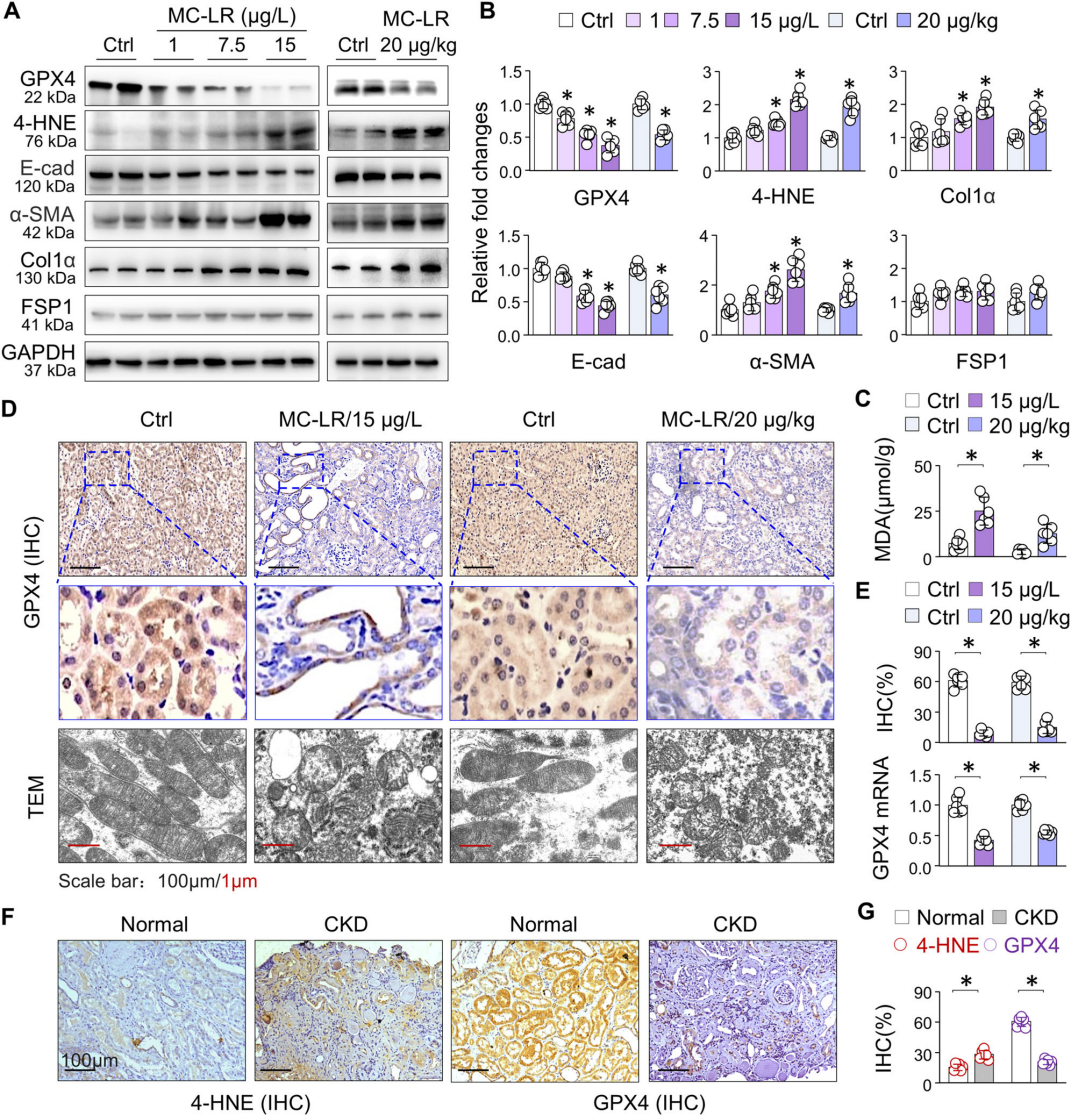
MC‐LR‐treated mice display renal GPX4 suppression, lipid peroxidation and ferroptotic mitochondrial alterations. A) Western blot analysis of GPX4, 4‐HNE, E‐cad, α‐SMA, Col1α and FSP1 in renal tissues from Ctrl and MC‐LR‐exposed mice. Two representative samples from each group are shown. B) Quantitation of (A). Data are presented as mean ± SD (one‐way ANOVA and Student's t‐test, *n* = 6). ^*^ *P* < 0.05 versus. Ctrl. C) MDA assay. Lipid peroxidation levels in renal tissues from Ctrl and MC‐LR‐exposed mice. Data are presented as mean ± SD (Student's t‐test, *n* = 6). ^*^ *P* < 0.05. D) Representative microscopic photographs of renal sections from Ctrl and MC‐LR‐exposed mice including GPX4 IHC staining and TEM. E) Quantitation of GPX4 IHC in (D) and qRT‐PCR analysis of GPX4 mRNA expression in renal tissues from Ctrl and MC‐LR‐exposed mice. Data are presented as mean ± SD (Student's t‐test, *n* = 6). ^*^ *P* < 0.05. F) Representative photomicrographs of kidney sections from renal patients (Normal and CKD) stained by IHC for 4‐HNE and GPX4. G) Quantitation of 4‐HNE and GPX4 IHC in (F). Data are presented as mean ± SD (Student's t‐test; *n* = 6). ^*^ *P* < 0.05.

To further elucidate the molecular mechanisms underlying MC‐LR‐induced kidney injury, we performed western blotting analysis on kidney tissues from both chronic and acute exposure groups. We observed marked upregulation of myofibroblast markers, including α‐smooth muscle actin (α‐SMA) and type I collagen (Col1α), alongside a significant reduction of the epithelial marker E‐cadherin (E‐cad) (**Figure** **2**A,B). In addition, the ferroptosis regulator GPX4 was markedly suppressed, coinciding with increased levels of lipid peroxidation markers 4‐hydroxynonenal (4‐HNE) (Figure 2A,B) and malondialdehyde (MDA) (Figure 2C). In contrast, the expression of another ferroptosis suppressor protein (FSP1),^[^ ^39^, ^40^ ^]^ which traps lipid peroxyl radicals independent of glutathione, was unaffected (Figure 2A,B). IHC staining further confirmed that GPX4, normally highly expressed in tubular epithelial cells, was significantly reduced following MC‐LR exposure (Figure 2D,E). Transmission electron microscopy (TEM) analysis revealed mitochondrial ferroptotic alterations including reduced mitochondrial size and loss of cristae (the bottom row in Figure 2D). Quantitative real‐time PCR (qRT‐PCR) analysis showed that GPX4 mRNA levels were significantly decreased after MC‐LR exposure, indicating that GPX4 suppression occurs primarily at the transcriptional level (Figure 2E). We also stained 6 renal biopsy samples from CKD patients with severe renal injury and found that comparing to 6 normal samples, the CKD renal sections showed increased 4‐HNE accumulated but much reduced GPX4 staining (Figure 2F,G), consistent with the mouse IHC staining patterns. Collectively, these results demonstrate that MC‐LR exposure suppresses GPX4 expression, promotes lipid peroxidation, and triggers tubular epithelium ferroptosis in mouse kidneys.

### 2.3 MC‐LR Induces *Gpx4* Promoter Hypermethylation and Selective Upregulation of DNMT1 and DNMT3a

**Figure 3:**
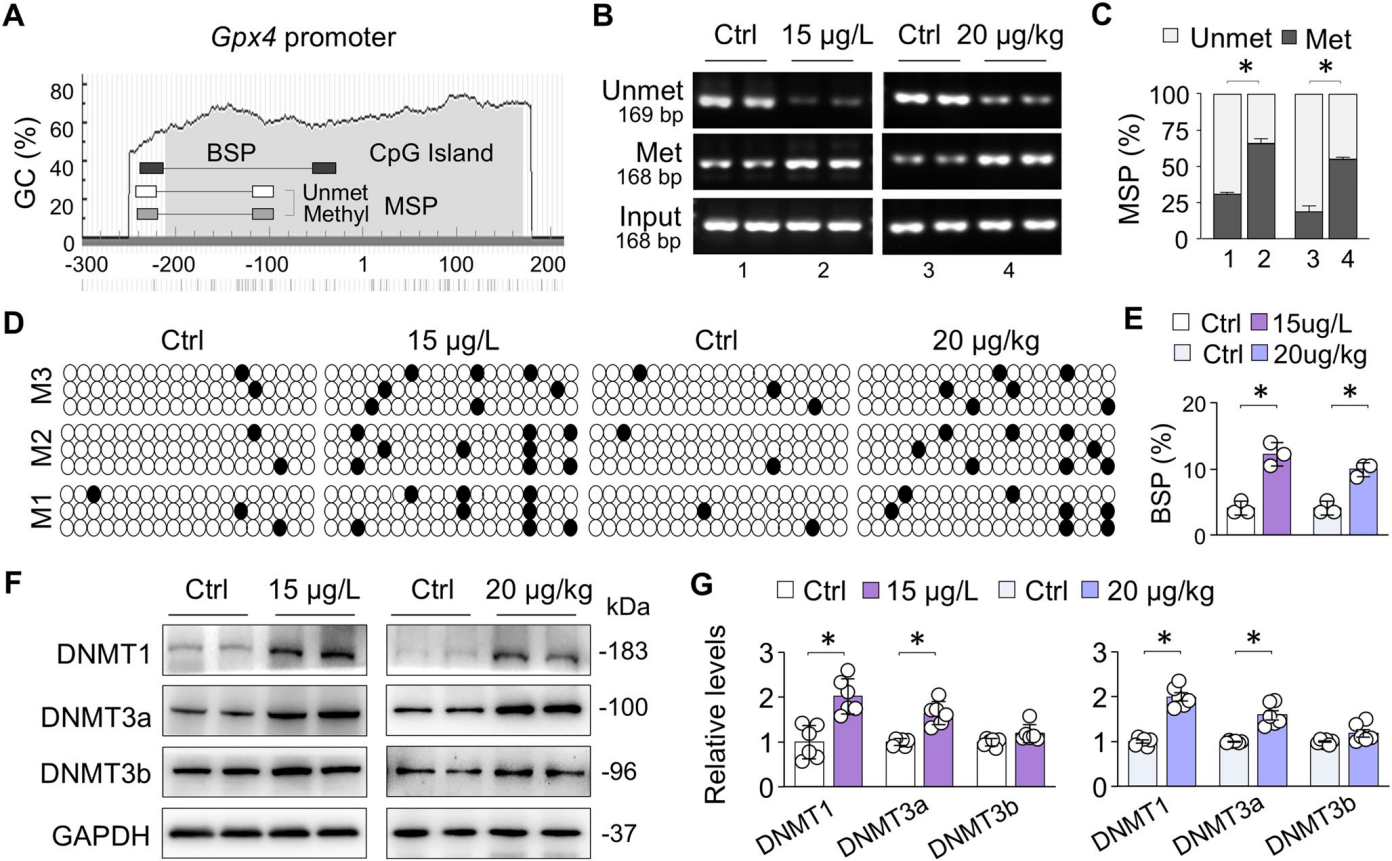
MC‐LR induces *Gpx4* promoter hypermethylation and selective upregulation of DNMT1 and DNMT3a. A) Schematic representation of the mouse *Gpx4* promotor, illustrating CpG island (grey area) and primer positions for MSP/BSP relative to the transcription start site (1). B) MSP analysis of *Gpx4* promotor methylation in the renal tissue from Ctrl and MC‐LR‐exposed mice. Representative agarose gel images show bands for methylated (Met), unmethylated (Unmet), and input PCR products. C) Quantitation of (B). Data are presented as mean ± SD after adjustment for input PCR products (Student's t‐test, *n* = 6). ^*^ *P* < 0.05. D) BSP analysis of CpG methylation in *Gpx4* promoter regions. Three mice from each group were randomly selected, and 3 clones (M1, M2, M3) from each mouse were sequenced. Each row represents a single clone, with each circle, open for unmethylated or black for methylated, indicating one CpG site. E) Quantitation of (D). Data are presented as mean ± SD (Student's t‐test). ^*^ *P* < 0.05. F) Western blot analysis of DNMT1, DNMT3a and DNMT3b expression in renal tissues from Ctrl and MC‐LR‐exposed mice. G) Quantitation of (F). Data are presented as mean ± SD (Student's t‐test, *n* = 6). ^*^ *P* < 0.05.

Using MethPrimer (http://www.urogene.org/methprimer), we identified a conserved CpG island located within the ‐210/+170 region relative to the transcription start site (TSS) of the mouse *Gpx4* promoter (**Figure** **3**A). Methylation specific PCR (MSP) revealed significantly increased methylation of this CpG island in renal tissues from both chronic and acute MC‐LR exposure groups (Figure 3B,C). To further validate these MSP findings, we performed bisulfite specific sequencing (BSP), a gold‐standard for DNA methylation assessment. The results confirmed increased methylation of *Gpx4* promoter following MC‐LR exposure (Figure 3D,E). As DNA methyltransferases play a central role in promoter methylation, we assessed DNMT expression in renal tissues. Western blotting showed significant upregulation of DNMT1 and DNMT3a following MC‐LR exposure, while DNMT3b expression remained largely unchanged (Figure 3F,G). These results indicate that MC‐LR induces selective upregulation of DNMT1 and DNMT3a, resulting in hypermethylation of the *Gpx4* promoter and GPX4 suppression.

### 2.4 MC‐LR Upregulates DNMT1 and DNMT3a via Inhibiting their Ubiquitin‐Mediated Degradation

**Figure 4:**
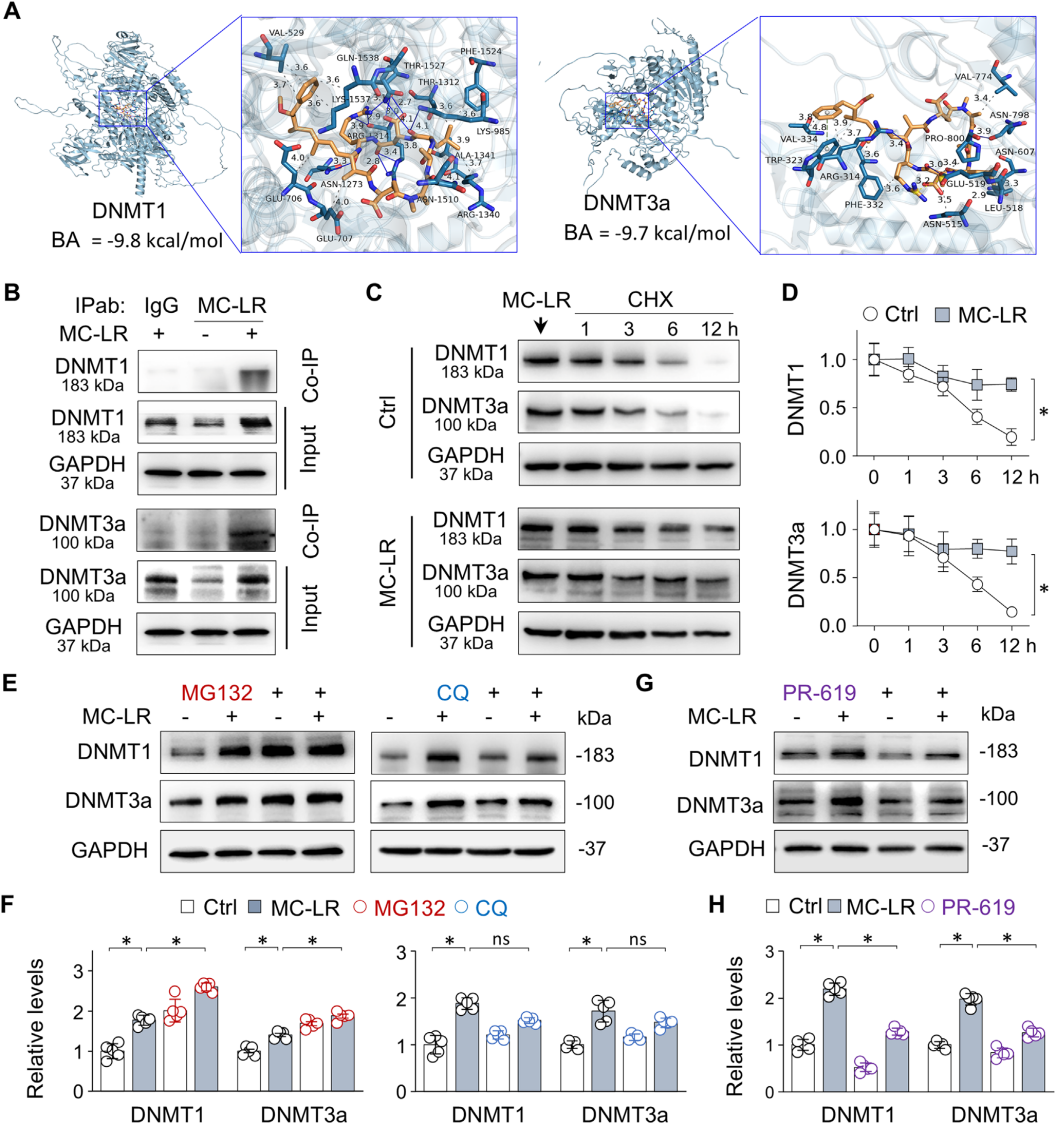
MC‐LR upregulates DNMT1 and DNMT3a via inhibiting their ubiquitin‐mediated degradation. A) Molecular docking analysis of MC‐LR interactions with DNMT1 or DNMT3a using AutoDock Vina to assess binding affinity (BA). B) Co‐IP analysis of renal tissues homogenates from Ctrl and MC‐LR‐exposed mice. IP was performed with either isotype‐matched immunoglobulin (IgG) or MC‐LR antibodies (IPab), and then immunoprecipitants (Co‐IP) and input tissue lysates (Input) were analyzed by western blotting for DNMT1 or DNMT3a levels. C) Western blot analysis of DNMT1 and DNMT3a levels in HK2 cells treated with MC‐LR first, and then with CHX (70 µm) for 1, 3, 6, or 12 h in the absence (Ctrl) or presence of MC‐LR. D) Quantitation of (C). Data were presented as mean ± SD (two‐way ANOVA, *n* = 5). ^*^ *P* < 0.05. E) Western blot analysis of DNMT1 and DNMT3a expression in HK2 cells treated with MC‐LR for 48h, and then with the presence or absence of MG132 (10 µm) for 6h or CQ (50 µm) for 12h. F) Quantitation of (E). Data were presented as mean ± SD (two‐way ANOVA, *n* = 5). ^*^ *P* < 0.05. G) Western blot analysis of DNMT1 and DNMT3a expression in HK2 cells treated with MC‐LR in the presence or absence of PR‐619 (10 µm) for 48h. H) Quantitation of (G). Data were presented as mean ± SD (two‐way ANOVA, *n* = 5). ^*^ *P* < 0.05.

To explore how MC‐LR upregulates DNMT1 and DNMT3a, we conducted molecular docking analysis using AutoDock Vina. MC‐LR is predicted to form hydrophobic interactions at VAL529, LYS1537, GLU706/707, ARG1314/1340, ALA1341, THR1312, and PHE1524, as well as hydrogen bonds at THR1527, ASN1510/1273, LYS985, ARG1314, and GLN1538 of DNMT1, with a binding affinity (BA) of ‐9.8 kcal mol^−1^. Additionally, MC‐LR is predicted to interact with DNMT3a through hydrophobic interactions at TRP323, ARG314, PHE332, VAL334/774, ASN515, LEU518, ASN607, and PRO800, along with hydrogen bonds at ARG314, GLU519, and ASN798, with a binding affinity of −9.7 kcal mol^−1^. These results suggest a strong interaction between MC‐LR and DNMT1/3a proteins (**Figure** **4**A). Co‐immunoprecipitation (Co‐IP) confirmed direct interaction between MC‐LR and DNMT1/3a (Figure 4B). Further protein stability assay using cycloheximide (CHX, a protein translation inhibitor) demonstrated that MC‐LR significantly slowed DNMT1 and DNMT3a degradation over time (12 h) (Figure 4C,D). Proteasome inhibition with MG132 increased DNMT1 and DNMT3a expression, while autophagy inhibition with chloroquine (CQ) had no effects (Figure 4E,F). Furthermore, treatment with the de‐ubiquitination inhibitor PR‐619 reversed the abnormal upregulation of DNMT1 and DNMT3a induced by MC‐LR (Figure 4G,H). These results indicate that MC‐LR directly binds to DNMT1 and DNMT3a and stabilizes them by inhibiting ubiquitination‐mediated proteasomal degradation.

### 2.5 MC‐LR‐Induced GPX4 Suppression is Coregulated by E2F4

**Figure 5:**
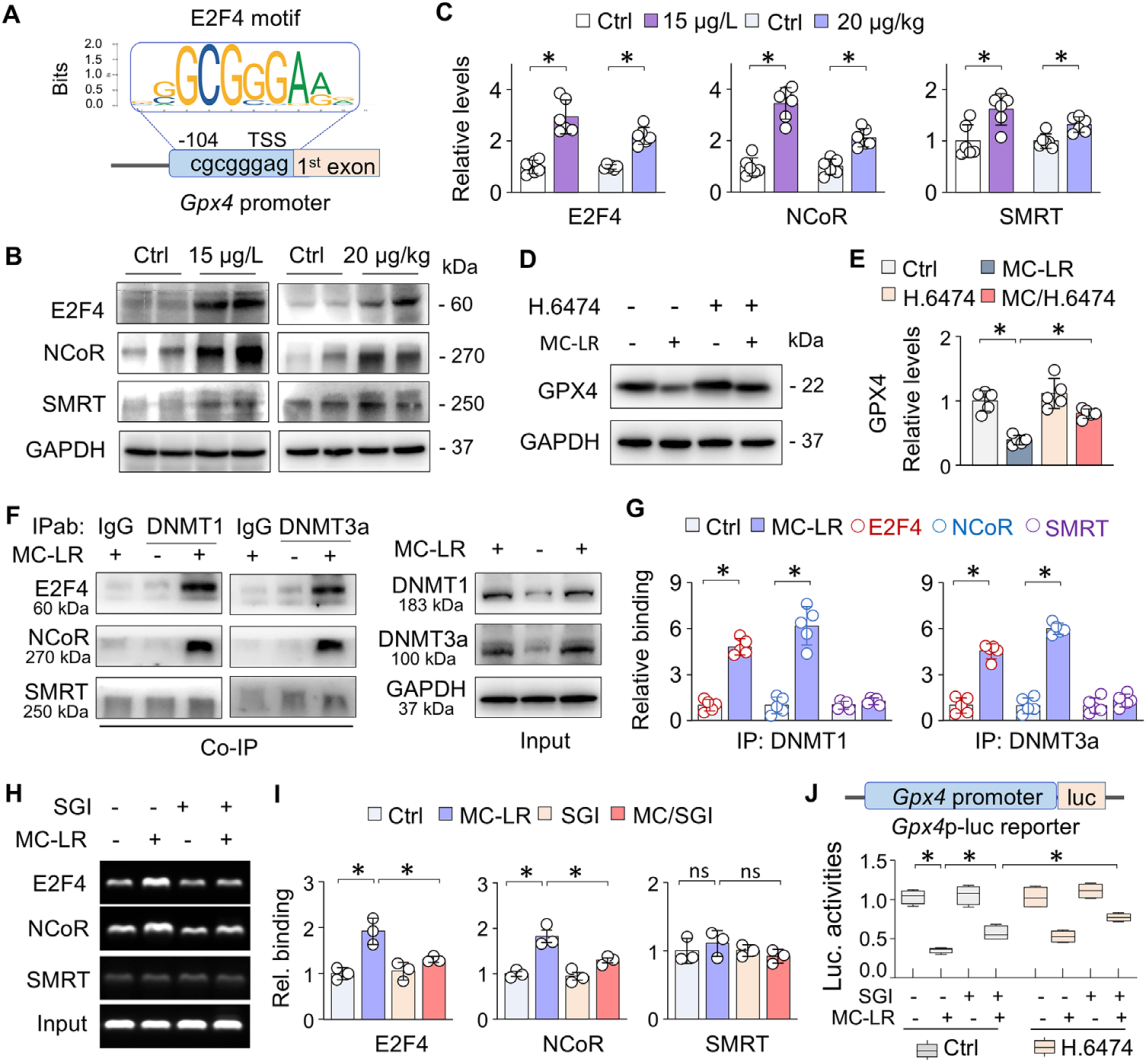
MC‐LR‐induced GPX4 suppression is coregulated by E2F4. A) Schematic representation of the E2F4 binding motif and its positions relative to the transcription start sites (TSS) in the mouse *Gpx4* promoter. B) Western blot analysis of E2F4, NCoR, and SMRT expression in renal tissues from Ctrl and MC‐LR‐exposed mice. C) Quantitation of (B). Data were presented as mean ± SD (Student's t‐test, *n* = 6). ^*^ *P* < 0.05. D) Western blot analysis of GPX4 in HK2 cells treated with MC‐LR (20 µm) in the presence or absence of E2F4 inhibitor HLM006474 (10 µm) for 48h. E) Quantitation of (D). Data were presented as mean ± SD (two‐way ANOVA, *n* = 5). ^*^ *P* < 0.05. F) Co‐IP analysis of renal tissues homogenates from Ctrl and MC‐LR‐exposed mice. IP was performed with either isotype‐matched immunoglobulin (IgG) or DNMT1/DNMT3a antibodies (IPab), and then immunoprecipitants (Co‐IP) and input tissue lysates (Input) were analyzed by western blotting for DNMT1 or DNMT3a levels. G) Quantitation of (F). Data were presented as mean ± SD (Student's t‐test, *n* = 5). ^*^ *P* < 0.05. H) ChIP assay of renal tissues from Ctrl and MC‐LR‐exposed mice with or without SGI‐1027 treatment. Immunoprecipitation was performed using antibodies against E2F4, NCoR, or SMRT, followed by PCR amplification of *Gpx4* promoter regions containing the E2F4 motif. Non‐immunoprecipitated DNA served as the input control (Input). PCR products were resolved on an agarose gel. I) Quantitation of (H). Data were presented as mean ± SD (two‐way ANOVA, *n* = 3). ^*^ *P* < 0.05. J) Luciferase reporter assay. HEK293 cells were transfected with a *Gpx4* promoter‐driven luciferase reporter (*Gpx4*p‐luc) and a renilla luciferase reporter control. Cells were treated with MC‐LR (20 µm) with or without SGI‐1027 (10 µm) in the absence or presence of HLM006474 (10 µm) for 48h. *Gpx4* promoter activity was normalized to renilla luciferase activity. Box‐and‐whisker plots represent 3 independent experiments (three‐way ANOVA). ^*^ *P* < 0.05.

To further explore the regulatory network responsible for GPX4 transcriptional suppression, we analyzed the proxy *Gpx4* promoter (500 bp) using JASPAR (https://jaspar.genereg.net). This analysis identified potential binding motifs for several transcription factors, including Krüppel‐like factor 5 (KLF5), specificity protein 1 (SP1), and E2F transcription factor 4 (E2F4). Among these, E2F4, a known transcriptional repressor associated with cell cycle arrest,^[^ ^41^ ^]^ exhibited a particularly high binding score at position ‐104 (sequence: cgcgggag; score: 11.985) close to the transcriptional starting site (TSS) (**Figure** **5**A). Western blotting analysis of renal tissues from MC‐LR‐ exposed mice showed a significant increase in E2F4 expression, accompanied by elevated levels of the transcriptional corepressors nuclear receptor corepressor (NCoR) and silencing mediator of retinoid and thyroid hormone receptor (SMRT) (Figure 5B,C).

To determine whether E2F4 play a functional role in regulating GPX4 expression, we treated HK2 cells with HLM006474, an E2F4 selective inhibitor. This treatment effectively alleviated the MC‐LR‐induced GPX4 suppression (Figure 5D,E). Co‐IP assay further confirmed that E2F4 and NCoR, but not SMRT, bound to DNMT1 and DNMT3a following MC‐LR exposure (Figure 5F,G). In addition, chromatin immunoprecipitation (ChIP) assay demonstrated that E2F4 and NCoR bound to the *Gpx4* promoter following MC‐LR exposure, which was significantly blocked by SGI‐1027, a DNMT inhibitor (Figure 5H,I). Consistent with these findings, luciferase reporter assay with a *Gpx4* promoter‐luciferase reporter plasmid demonstrated that MC‐LR‐induced transcriptional repression of the *Gpx4* promoter was significantly reversed by SGI‐1027, with HLM006474 providing additional relief (Figure 5J). Together, these results indicate that MC‐LR‐induced transcriptional repression of GPX4 is orchestrated by the coordinated action of DNMT1/3a, E2F4, and NCoR, highlighting a complex epigenetic and transcriptional regulatory mechanism.

### 2.6 DNMT Inhibition Alleviates MC‐LR‐Induced GPX4 Suppression and Lipid Peroxidation in Renal Tubular Epithelial Cell

**Figure 6:**
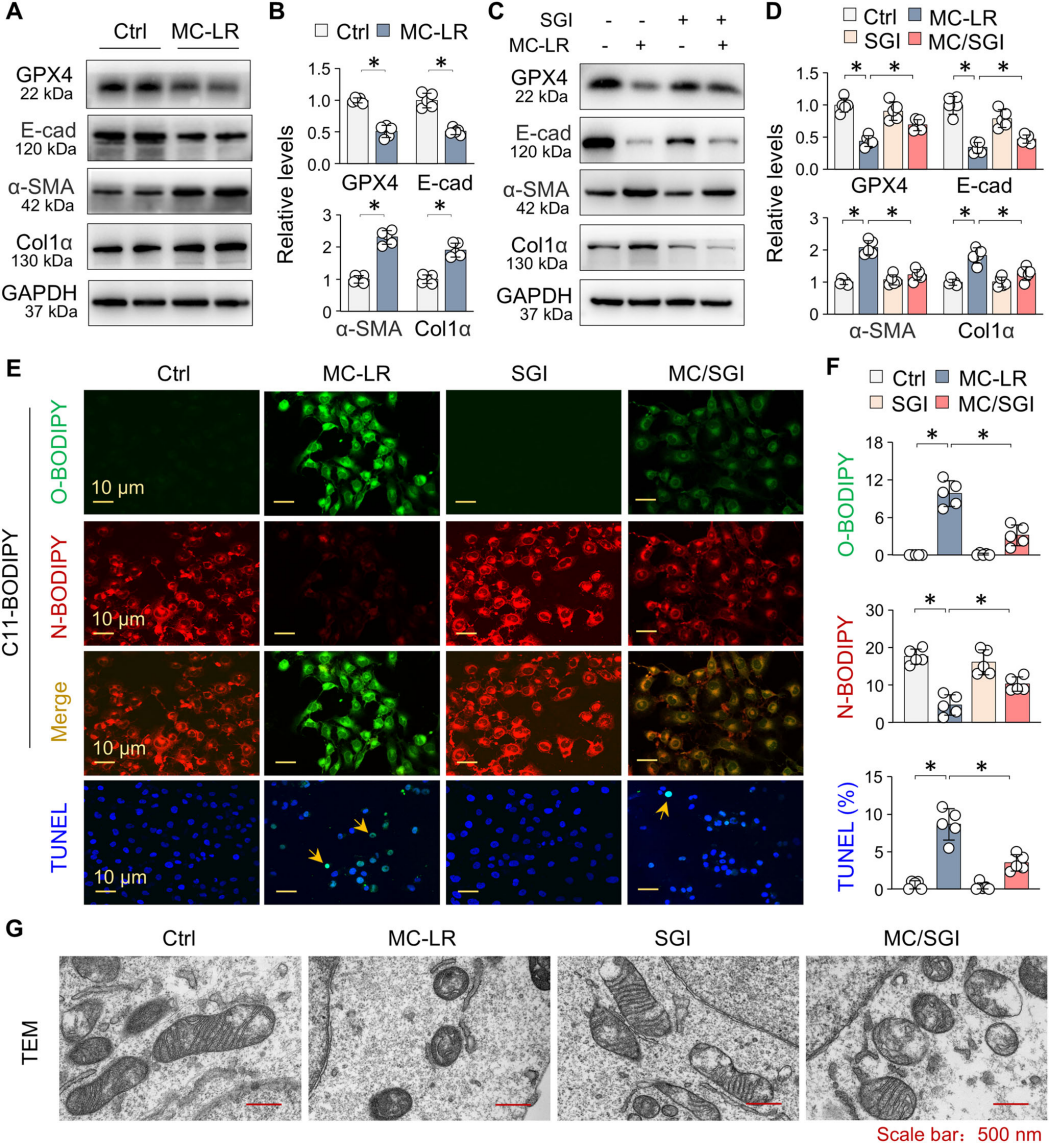
DNMT inhibition alleviates MC‐LR‐induced GPX4 suppression and lipid peroxidation in renal tubular epithelial cell. A) Western blot analysis of GPX4, E‐cad, α‐SMA, and Col1α expression in HK2 cells treated with MC‐LR (20 µm) for 48h. B) Quantitation of (A). Data are presented as mean ± SD (Student's t‐test, *n* = 5). ^*^ *P* < 0.05. C) Western blot analysis of GPX4, E‐cad, α‐SMA, and Col1α expression in HK2 cells treated with MC‐LR with or without SGI‐1027 (10 µm) for 48h. D) Quantitation of (C). Data are presented as mean ± SD (two‐way ANOVA, *n* = 5). ^*^ *P* < 0.05. E) Representative images of C11‐BODIPY and TUNEL staining in HK2 cells treated with MC‐LR in the presence or absence of SGI‐1027 for 48h. C11‐BODIPY appeared red for non‐oxidized (N‐) and green for oxidized (O‐) BODIPY, respectively, with yellow indicating overlap. TUNEL‐positive cells are indicated by arrows. F) Quantitation of (E). Data from five repeated experiments are presented as the mean percentage of positive cells ± SD (two‐way ANOVA). ^*^ *P* < 0.05. G) HK2 cells treated as above were examined by TEM.

To confirm that the DNMT‐associated ferroptotic and fibrotic alterations induced by MC‐LR occur in renal tubule epithelial cells, we treated HK2 cells with MC‐LR. Western blotting analysis revealed that MC‐LR treatment significantly decreased the expression of GPX4 and E‐cad, while markedly increasing the expression of α‐SMA and Col1α (**Figure** **6**A,B). To determine whether DNMT dysregulation contributes to these alterations, we treated MC‐LR‐treated HK2 cells with SGI‐1027. SGI‐1027 treatment effectively reversed the MC‐LR‐induced downregulation of GPX4 and E‐cad, while also reducing the upregulation of α‐SMA and Col1α (Figure 6C,D).

Next, we assessed lipid oxidation and ferroptotic changes in HK2 cells using the fluorescent probe C11‐BODIPY, a lipid peroxidation sensor, and TUNEL staining. In control cells, non‐oxidized BODIPY (N‐BODIPY, red signal, upper three panels in Figure 6E) was predominantly detected, with minimal TUNEL‐positive staining (lower panel in Figure 6E). In contrast, MC‐LR triggered significant accumulation of oxidized BODIPY (O‐BODIPY, green signal) and a marked increase in TUNEL‐positive cells. Notably, co‐treatment with SGI‐1027 effectively reduced both oxidized BODIPY levels and TUNEL‐positive cell counts (Figure 6E,F). In addition, TEM showed that MC‐LR caused ferroptotic mitochondrial changes such as small mitochondria and diminished crista. However, SGI‐1027 effectively corrected these alterations (Figure 6G). Collectively, these findings support that MC‐LR induction of DNMT1/3a suppresses GPX4 expression, promoting lipid peroxidation, and ferroptotic and fibrotic alteration in tubular epithelial cells.

### 2.7 DNMT Inhibition Alleviates MC‐LR‐Induced Renal Ferroptosis and Fibrotic Damage

**Figure 7:**
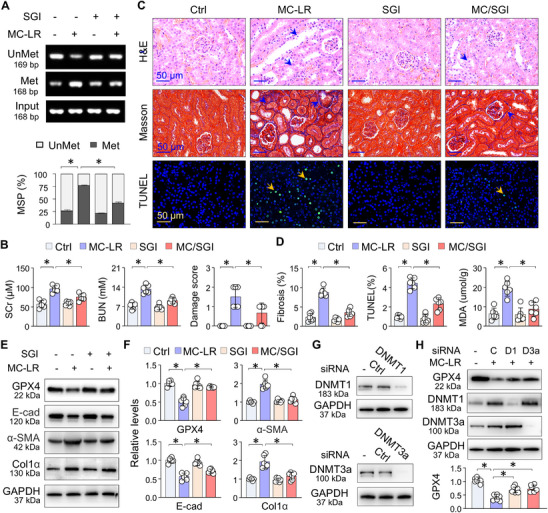
DNMT inhibition alleviates MC‐LR‐induced renal ferroptosis and fibrotic damage. A) MSP assay. Representative agarose gel images showing methylated (Met), unmethylated (Unmet) and Input PCR products. Below, quantitative analysis of the agarose gel bands. Data are presented as mean ± SD adjusted for Input PCR products (two‐way ANOVA, *n* = 6). ^*^ *P* < 0.05. B) The levels of SCr and BUN and quantification of tubular damage from Ctrl and MC‐LR‐exposed mice treated with or without SGI‐1027 (2.5 mg kg^−1^) for two weeks. Data are presented as mean ± SD (two‐way ANOVA, *n* = 6). ^*^ *P* < 0.05. C) Representative microscopic images of renal sections from Ctrl and MC‐LR‐exposed mice treated with or without SGI‐1027. Staining includes H&E, Masson's trichrome, and TUNEL. Arrows indicate tubular damage, collagen deposition and TUNEL‐positive cells. D) Quantitation of (C) and MDA assay results. Data are presented as mean ± SD (two‐way ANOVA, *n* = 6). ^*^ *P* < 0.05. E) Western blot analysis of GPX4, E‐cad, α‐SMA and Col1α expression in renal tissues from Ctrl and MC‐LR‐exposed mice treated with or without SGI‐1027. F) Quantitation of (E). Data are presented as mean ± SD (two‐way ANOVA, *n* = 6). ^*^ *P* < 0.05. G) Western blotting. HK2 cells were transfected with siRNA‐ control(C), siRNA‐DNMT1(D1) or siRNA‐DNMT3a (D3a) for 24h, and assayed for DNMT1 and DNMT3a, respectively. H) Western blotting. The siRNA‐transfected HK2 cells were treated with or without MC‐LR for 48h, and then the cell lysates were assayed for GPX4, DNMT1 and DNMT3a. Quantification of GPX4 below the blots was presented as mean ± SD (two‐way ANOVA, *n* = 6). ^*^ *P* < 0.05.

To confirm that MC‐LR‐induced DNMT1/3a upregulation, *Gpx4* promoter hypermethylation and GPX4 suppression play a functional role in renal ferroptosis/fibrotic damage in vivo, we treated MC‐LR‐exposed mice with SGI‐1027. As expected, MSP analysis confirmed that SGI‐1027 treatment significantly reduced hypermethylation of the *Gpx4* promoter induced by MC‐LR (**Figure** **7**A). In parallel, serum levels of creatinine and urea nitrogen and histological analysis demonstrated that SGI‐1027 effectively alleviated MC‐LR‐induced tubular damage, collagen deposition, and cell death (Figure 7B–D). Additionally, SGI‐1027 significantly decreased the levels of MDA (Figure 7D). Western blotting analysis further validated that SGI‐1027 reversed the MC‐LR‐induced deregulations of GPX4, E‐cad, α‐SMA, and Col1α expression (Figure 7E,F). To confirm the specific role of DNMT1 and DNMT3a isoform in inducing GPX4 suppression, we designed siRNAs specifically targeting DNMT1, DNMT3a (Figure 7G) and found that knockdown of DNMT1, DNMT3a each significantly reversed MC‐LR‐induced GPX4 suppression (Figure 7H), suggesting DNMT1 and DNMT3a contribute to ferroptotic GPX4 suppression in renal tubular damage. These results suggest that MC‐LR‐induced DNMT1/3a upregulation, *Gpx4* promoter hypermethylation and GPX4 suppression causally affect renal ferroptosis and fibrotic damage. More importantly, pharmacological DNMT inhibition is effective to mitigate the pathologies.

### 2.8 Ferroptosis Inhibition Alleviates MC‐LR Nephrotoxicity

**Figure 8:**
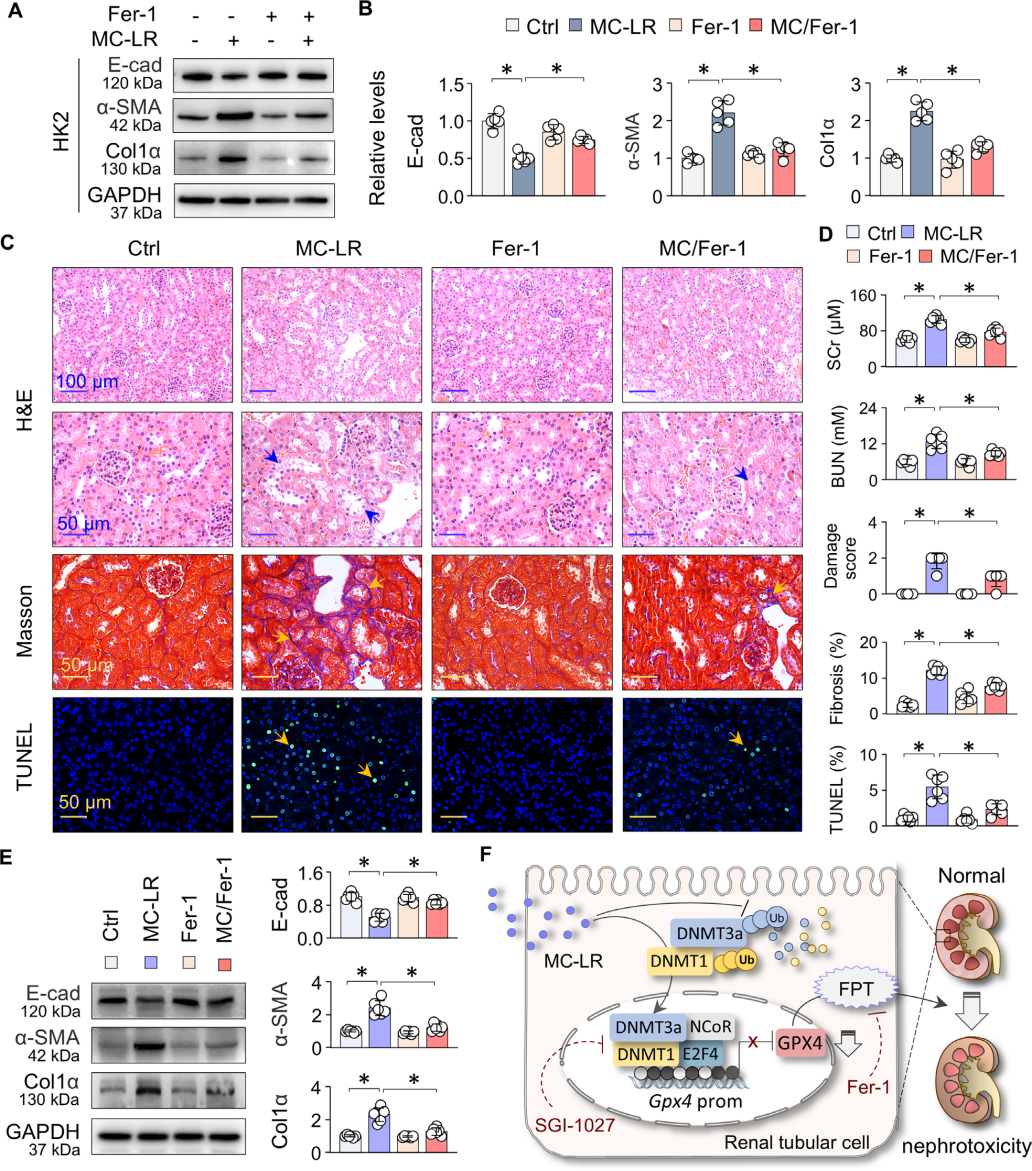
Ferroptosis inhibition alleviates MC‐LR nephrotoxicity. A) Western blot analysis of E‐cad, α‐SMA, and Col1α in HK2 cells untreated or treated with MC‐LR, with or without Fer‐1 (1 µm) for 48h. B) Quantitation of (A). Data are presented as mean ± SD (two‐way ANOVA, *n* = 5). ^*^ *P* < 0.05. C) Ctrl and MC‐LR‐exposed mice were treated with or without Fer‐1(5 mg kg^−1^) for two weeks. Representative microscopic images of renal sections. Staining includes H&E, Masson's trichrome, and TUNEL. Arrows indicate tubular damage, collagen deposition, and TUNEL‐positive cells. D) The levels of SCr and BUN and quantification of (C) from Ctrl and MC‐LR‐exposed mice treated with or without Fer‐1. Data are presented as mean ± SD (two‐way ANOVA, *n* = 6). ^*^ *P* < 0.05. E) Western blot analysis and quantitation of E‐cad, α‐SMA, and Col1α expression in renal tissues from Ctrl and MC‐LR‐exposed mice with or without Fer‐1 treatment. Data are presented as mean ± SD (two‐way ANOVA, *n* = 6). ^*^ *P* < 0.05. F) A schematic diagram illustrating MC‐LR‐Induced renal injury. MC‐LR increases DNMT1/3a levels by preventing their ubiquitin (Ub)‐mediated proteasomal degradation, leading to hypermethylation of the *Gpx4* promoter. This promotes the formation of a transcriptional repressive complex comprising DNMT1/3a, E2F4, and NCoR, thereby suppressing GPX4 transcriptional, triggering tubular epithelial ferroptosis (FPT) and renal fibrotic injuries (solid lines). Conversely, inhibition of DNMT (SGI‐1027) and ferroptosis (Fer‐1) effectively disrupts this pathological cascade (dashed lines).

To further confirm that ferroptosis plays a central role in MC‐LR‐induced renal fibrotic damage, we administered Ferrostatin‐1 (Fer‐1), a selective ferroptosis inhibitor, to both MC‐LR‐exposed HK2 cells and mice. In HK2 cells, Fer‐1 treatment significantly reversed the dysregulation of E‐cad, α‐SMA, and Col1α induced by MC‐LR (**Figure** **8**A,B). In MC‐LR‐exposed mice, Fer‐1 treatment effectively mitigated serum levels of creatinine and urea nitrogen, tubular damage, collagen deposition, and ferroptotic cell counts (Figure 8C,D). Western blotting analysis further demonstrated that Fer‐1 attenuated the MC‐LR‐induced abnormal expression of E‐cad, α‐SMA, and Col1α (Figure 8E). Together, these results underscore that ferroptosis is a major driver of MC‐LR‐induced renal injury, and targeting ferroptosis with Fer‐1 represents a promising therapeutic strategy to protect against MC‐LR nephrotoxicity.

## 3 Discussion

Emerging evidence indicates that MC‐LR exposure affects multiple organs, including the liver, kidneys, heart, lungs, testes, and brain, through diverse molecular mechanisms.^[^ ^42^ ^]^ In this study, we demonstrate that MC‐LR directly binds to and upregulates DNMT1 and DNMT3a, likely by inhibiting their ubiquitin‐mediated proteasomal degradation. This leads to hypermethylation of the *Gpx4* promoter and the formation of a repressive complex comprising DNMT1/3a, E2F4 and NCoR, resulting in GPX4 transcriptional suppression. These epigenetic modifications trigger ferroptosis in renal tubular epithelial cells that mediates MC‐LR nephrotoxicity. Notably, inhibition of DNMT1/3a and ferroptosis effectively disrupts the process and mitigates MC‐LR‐induced renal pathologies (Figure 8F). Therefore, our findings unveil a novel molecular pathway linking MC‐LR‐induced DNA methylation to ferroptosis and nephrotoxicity, offering potential therapeutic targets for intervention.

Regulated cell death plays a critical role in MC‐LR‐induced cytotoxicity. For instance, MC‐LR induces the activation of the NACHT, LRR and PYD domains‐containing protein 3 (NLRP3) inflammasome by promoting forkhead box protein O1 (FOXO1) hyperphosphorylation, triggering pyroptosis in mouse hepatocytes and contributing to hepatotoxicity.^[^ ^43^ ^]^ In rat testicular Sertoli cells, MC‐LR induces mitochondrial‐mediated apoptosis by activating the caspases pathway and increasing reactive oxygen species production, leading to reproductive toxicity.^[^ ^44^ ^]^ Additionally, MC‐LR exposure causes mitochondrial structural damage and downregulation of GPX4 and solute carrier family 7 member 11 (SLC7A11), resulting in ferroptosis‐like changes in mouse brain.^[^ ^45^ ^]^ In this study, we provide the first evidence that both chronic and acute MC‐LR exposure suppresses GPX4, leading to renal ferroptosis. Ferroptotic changes were primarily observed in renal tubular epithelial cells, as indicated by elevated levels of 4‐HNE, MDA, lipid peroxidation and mitochondrial structure deterioration. More importantly, treatment with the ferroptosis inhibitor Fer‐1 effectively prevented MC‐LR‐induced renal fibrotic damage, highlighting the crucial role of tubular epithelial ferroptosis in MC‐LR‐induced nephrotoxicity.

MC‐LR exerts its cytotoxic effects primarily by inhibiting protein phosphatase PP1 and PP2A,^[^ ^46^ ^]^ thereby disrupting key transcriptional regulators and signaling pathways/molecules, such as cellular tumor antigen p53, mitogen‐activated protein kinases (MAPKs) and calcium‐calmodulin‐dependent protein kinase II (CaMKII).^[^ ^43^ ^]^ However, increasing evidence suggests that MC‐LR interacts with multiple proteins, influencing diverse cellular processes. A study using thermal proteome profiling and SWATH‐DIA mass spectrometry identified 129 proteins that directly interact with MC‐LR in human neuroblastoma cells.^[^ ^47^ ^]^ In a mouse model of pulmonary fibrosis, MC‐LR binds to G Protein‐Coupled Receptor 78 (GRP78), modulating macrophage M2 polarization and suppressing the endoplasmic reticulum stress response.^[^ ^48^ ^]^ Additionally, MC‐LR interacts with extracellular regulated protein kinases 2 (ERK2), reducing dopamine levels by enhancing heat shock cognate protein HSC70,^[^ ^49^ ^]^ In a xenografted rat model of hepatocellular carcinoma, MC‐LR promotes DNA methylation at the homeobox protein aristaless‐like 4 (ALX4) promoter, and the subsequent transcriptional repression of ALX4 contributes to tumor progression.^[^ ^50^ ^]^ Consistent with its role in DNA methylation, our findings reveal that MC‐LR directly binds to DNMT1 and DNMT3a, stabilizing them by preventing ubiquitin‐mediated degradation. This leads to *Gpx4* promoter hypermethylation, GPX4 transcriptional suppression and epithelial ferroptosis effects that were effectively reversed by DNMT inhibition. Thus, our study identifies MC‐LR‐induced DNMT1/3a stabilization as a novel epigenetic mechanism contributing to kidney damage, providing new insights into its pharmacological properties, pathological effects, and potential therapeutic targets.

Transcriptional repression mediated by DNA methylation involves multiple factors beyond DNA methylation‐modifying enzymes, particularly transcriptional repressors and co‐repressors. While these proteins do not directly methylate DNA, they are crucial for recruiting and stabilizing DNMTs and chromatin remodelers at specific gene loci. Their coordinated actions establish a transcriptionally repressive chromatin state, leading to sustained gene silencing.^[^ ^51^, ^52^ ^]^ For example, the transcription factor KLF5 and corepressor NCoR contribute to DNA methylation‐mediated GPX4 repression in osteolytic and osteoporosis bone.^[^ ^53^, ^54^ ^]^ In MC‐LR‐exposed kidney, we found that the transcription repressor E2F4 and corepressor NCoR, but not SMRT, a related corepressor, are upregulated and bind to the *Gpx4* promoter. While E2F4 is a well‐known transcription repressor,^[^ ^41^ ^]^ its role and targets under ferroptotic conditions remain unclear. Our findings showed that inhibiting E2F4 effectively attenuated GPX4 suppression, highlighting its role in MC‐LR‐induced gene silencing. We propose that following MC‐LR exposure, DNMT1/3a, E2F4 and NCoR form a transcriptional repressive complex at the *Gpx4* promoter, preventing DNA demethylation and ensuring heritable GPX4 repression. This epigenetic silencing mechanism likely contributes to renal ferroptotic and fibrotic damage. However, further investigation is needed to elucidate the precise regulatory mechanisms, particularly the involvement of additional transcriptional regulators and the specificity of methylation sites in MC‐LR‐induced GPX4 suppression.

## 4 Conclusion

In conclusion, our study identifies a novel molecular mechanism by which MC‐LR promotes nephrotoxicity through inhibition of DNMT degradation, *Gpx4* promoter hypermethylation and transcriptional suppression, and ferroptosis induction. These findings provide new insights into how environmental toxins influence epigenetic regulation and regulated cell death pathways, shaping disease pathogenesis at the molecular level. Furthermore, our work highlights potential therapeutic strategies targeting epigenetic modifications and ferroptosis for the prevention and treatment of MC‐LR‐induced cytotoxicity in the kidneys and potentially other organs.

## 5 Experimental Section

### Ethics Statement

All animal experiments were approved by the Institutional Animal Care and Use Committee of Drum Tower Hospital Affiliated to Nanjing University Medical School (Approval No. 2023AE01008) and conducted in accordance with the ARRIVE guidelines and the European Directive 2010/63/EU.

Six human kidney tissue samples were obtained from CKD patients (aged 45‐65 years) undergoing diagnostic renal biopsy at Danyang People's Hospital, Jiangsu, China. The normal samples were paracancerous tissues from renal cancer patients underwent tumor‐removal surgery. The study was approved by the hospital ethics committee (Approval No. 20230512), and written informed consent was obtained from all participants.

### Animal Models

Seven‐week‐old male BALB/c mice were purchased from the GemPharmatech Co., Ltd. Mice were housed in a specific pathogen‐free (SPF) environment under a 12 h light‐dark cycle, with controlled temperature and humidity, and provided food and water ad libitum. For the chronic exposure experiment, mice were randomly divided into four groups (*n* = 6 per group) and given drinking water containing different concentrations of MC‐LR (0, 1, 7.5, 15 µg L^−1^; E‐LR‐M001, Taiwan Algal Science Inc., purity>95%) for 12 months.^[^ ^55^ ^]^ For the acute exposure experiment, mice were randomly allocated into two groups (*n* = 6 per group). The exposure group received daily intraperitoneal injections of MC‐LR (20 µg kg^−1^·bw) for 14 consecutive days,^[^ ^7^ ^]^ while the control group received equivalent volume of normal saline. For intervention experiments, mice were randomly allocated into four groups (n = 6 per group): (1) vehicle control; (2) MC‐LR model: daily intraperitoneal injection of MC‐LR (20 µg kg^−1^·bw); (3) SGI‐1027 or Fer‐1: intraperitoneal injection on alternate days of SGI‐1027 (HY‐13962, MCE, USA; 2.5 mg kg^−1^) or Fer‐1 (HY‐100579, MCE; 5 mg kg^−1^); (4) SGI‐1027 or Fer‐1 intervention: treatment with SGI‐1027 or Fer‐1 began one day after MC‐LR administration. Following the final treatment, mice were euthanized via intraperitoneal injection of sodium pentobarbital (240 mg kg^−1^). Blood samples and kidneys tissues were harvested for further assay. Serum creatinine and blood urea nitrogen levels were measured using a Hitachi LabOSPECT 008AS automated biochemical analyzer (Hitachi High‐Tech Corporation, Tokyo, Japan) with the respective Creatinine FS and Urea FS kits, strictly following the manufacturer's protocols.

### Hematoxylin and Eosin (H&E), Masson's Trichrome and Immunohistochemistry (IHC) Staining

Renal tissue sections were prepared and stained with H&E, Masson's trichrome, and immunohistochemical markers following established protocols.^[^ ^55^ ^]^ Tubular damage was evaluated by H&E staining and scored from 0 to 4 based on brush border loss, epithelial swelling and vacuolization, epithelial cell detachment, tubule dilatation, tubule atrophy: 0 (no damage or <10%), 1 (10%–24%), 2 (25%–49%), 3 (50%–75%), 4 (>75%).^[^ ^22^, ^56^ ^]^ Fibrosis was assessed by Masson's trichrome staining, quantified as the percentage of collagen ‐ positive area relative to the total renal area using ImageJ software. IHC staining intensity for MC‐LR (ALX‐804‐320, Enzo Life Science, Switzerland), GPX4 (A25009, ABclonal, China), and 4‐HNE (ab48506, Abcam, UK) was also quantified with ImageJ software. Images were obtained using a Leica DM2500 microscope.

### Perl's Prussian Blue Staining

Perl's Prussian blue staining was used to detect hemosiderin accumulation in renal sections with a commercial kit (G1029, Servicebio, China), incorporating 3,3′‐diaminobenzidine (DAB) for signal enhancement. Potassium ferrocyanide was applied to release trivalent iron from proteins, forming blue, water‐insoluble ferrocyanide precipitates. DAB was then added to amplify the signal, producing a brown compound. The area of brown staining, representing iron deposition, was quantified as a percentage of the total tissue area using ImageJ.

### Terminal Deoxynucleotidyl Transferase dUTP Nick‐End Labeling (TUNEL) Staining

TUNEL staining were performed on kidney tissue sections and HK2 cells using a Fluorescein (FITC) TUNEL Detection Kit (G1501, Servicebio) following the manufacturer's protocol. Kidney tissues were fixed in 4% paraformaldehyde, embedded in paraffin, sectioned, dewaxed and rehydrated. HK2 cells were fixed on slides with 4% paraformaldehyde the percentage of TUNEL‐positive cells was calculated, and images were captured using an Olympus FV3000 confocal fluorescence microscope.

### Transmission Electron Microscopy (TEM)

TEM was performed by Servicebio (China). Freshly dissected renal tissue and cells after various treatments were fixed in aldehyde solution at room temperature for 2 h, and then stored at 4 °C. Samples were dehydrated, embedded, sectioned, and examined under a transmission electron microscopy.

### Cell Culture and Treatment

HK2 (human renal tubular epithelial) and HEK293 (human embryonic kidney) cells (ATCC, USA) were cultured in DMEM/F12 (C11330500BT, Gibco, USA) or DMEM (C11995500BT, Gibco), respectively, supplemented with 10% fetal bovine serum (FBS; SA211.02, CellMax, China) and 1% penicillin/streptomycin (15140‐122, Gibco) in a humidified 5% CO_2_ incubator at 37 °C. Cells were treated with various agents, including MC‐LR, SGI‐1027, Fer‐1, CHX (HY‐12320, MCE), CQ (HY‐17589A, MCE), MG132 (HY‐13259, MCE), PR‐619 (HY‐13814, MCE), and HLM006474 (HY‐16667, MCE) as specified.

### Western Blotting

Western blotting was performed on mouse renal tissues and cell lysates according to previously established protocols.^[^ ^55^ ^]^ A list of all antibodies used in this study is provided in the supplementary information (Table S1, Supporting Information). After incubation with primary antibodies, membranes were washed and incubated with HRP‐conjugated secondary antibodies: goat anti‐rabbit IgG (FDR007, FDbio, China) or goat anti‐mouse IgG (FDM007, FDbio). Protein bands were visualized using ECL detection reagents (P10300, New Cell & Molecular Biotech, China), and imaged with a chemiluminescent detection system (Tanon, China). Band intensities were quantified using Image J software.

### Quantitative Real‐Time PCR (qRT‐PCR)

Total RNA was extracted from renal tissues using the FastPure Cell/Tissue Total RNA Isolation Kit (RC101‐01, Vazyme, China). Equal amounts of mRNA were reverse‐transcribed into cDNA using the HiScript RT SuperMix kit (R122‐01; Vazyme). qRT‐PCR was performed using ChamQ Universal SYBR qPCR Master Mix (Q711‐02; Vazyme) on a CFX96 Deep Well real‐time PCR system (Biorad, USA). A list of all primer sequences used in this study is provided in the supplementary information (Table S2, Supporting Information). Cycle threshold (Ct) values were recorded, and relative gene expression was calculated using the 2^−ΔΔCt^ method.

### Malondialdehyde (MDA) Assay

MDA levels in renal tissues were measured using the Lipid Peroxidation MDA Assay Kit (S0131S, Beyotime), following the manufacturer's instructions. Tissue homogenates were mixed with MDA working solution and incubated at 100 °C for 15 min. Absorbance was measured at 532 nm using a multifunctional microplate reader (Molecular Devices M3, USA).

### Methylated Specific PCR (MSP) and Bisulfite Sequencing PCR (BSP)

The *Gpx4* promoter region was analyzed using MethPrimer online software (http://www.urogene.org/methprimer)^[^ ^57^ ^]^ to identify CpG islands and design primers for MSP and BSP. Genomic DNA was extracted from kidney tissue using the Tissue/Cell Genomic DNA Extraction Kit (DP304‐02, TIANGEN, China) and modified with the DNA Bisulfite Conversion Kit (DP215‐02, TIANGEN) per manufacturer's guidelines. MSP was performed using methylated primers: M‐*Gpx4*F/ M‐*Gpx4*R (169 bp, −246/−78); unmethylated primers, U‐*Gpx4*F/ U‐*Gpx4*R (168 bp, −244/−77), and input DNA control primers: Inp‐*Gpx4*F/Inp‐*Gpx4*R (169 bp) (Table S2, Supporting Information). PCR products were separated on 1.5% agarose gels. Densitometry was performed with ImageJ to quantify amplicons. For BSP, the bisulfite‐converted genomic DNA from mouse renal tissues was amplified using primers B‐*Gpx4*F and B‐*Gpx4*R (Table S2, Supporting Information).^[^ ^53^ ^]^ PCR products were gel‐purified, cloned into the pGEM‐T Easy Vector (A1360; Promega, USA), and sequenced. For each sample, three colonies were randomly selected for sequencing, and methylation levels were calculated as the ratio of methylated cytosines to total cytosines within the cloned fragment.

### Molecular Docking

Protein structures of for DNMT1 (UniProt ID: P13864) and DNMT3a (UniProt ID: O88508) were obtained from the Protein Data Bank (https://www.pdb.org/), and the molecular structure of MC‐LR was downloaded from PubChem (https://pubchem.ncbi.nlm.nih.gov/). Docking simulations between MC‐LR and DNMT1/DNMT3a was performed using the AutoDock Vina program (Vina, version 1.1.2).^[^ ^58^ ^]^ Ligand preparation – including hydrogen addition, charge verification, and rotatable bond assignment – was carried out using AutoDockTools (http://mgltools.scripps.edu/downloads). Docking results were evaluated by binding affinity scores. Molecular interactions were visualized in 3D representations using PyMOL.

### RNA Interferences

DNMT1 and DNMT3a knockdown in HK2 cell were performed with small interference RNA (siRNA) as established protocol. A scrambled RNA of DNMT1 gene (sense oligo: UCGACCUGGUUUGAUACUUAUTT dTdT; antisense oligo: AUAAGUAUCAAACCAGGUCGATT dTdT) and DNMT3a gene (sense oligo: GCGUCACACAGAAGCAUAUTT dTdT; antisense oligo: AUAUGCUUCUGUGUGACGCTT dTdT) were produced by GenScript Biotech Co., and cells were transfected with siRNA for 24 h followed by MC‐LR treatment for 48 h.

### Co‐Immunoprecipitation (Co‐IP)

Renal tissue lysates were incubated overnight with antibodies to MC‐LR, DNMT1, DNMT3a, or with isoform‐matched IgG controls. Immunocomplexes were captured using Protein A+G Agarose (P2055, Beyotime), followed by washing.^[^ ^59^ ^]^ Western blotting was then performed on the immunoprecipitation using DNMT1, DNMT3a, E2F4, NCoR, or SMRT antibody (Table S1, Supporting Information). Non‐immunoprecipitated lysates served as loading controls.

### Chromatin Immunoprecipitation (ChIP)

ChIP assays on renal tissues were performed using a commercial kit (P2078, Beyotime, China).^[^ ^53^ ^]^ Samples were immunoprecipitated overnight at 4 °C with antibodies against E2F4, NCoR, or SMRT (Table S1, Supporting Information). PCR amplification was carried out using primers (Table S2, Supporting Information), targeting the proximal Gpx4 promoter region (−239 to −14), which contains a putative E2F4 binding motif (−104/cgcgggag). PCR products were resolved on 1.5% agarose gels and visualized under UV light. Band intensities were quantified with ImageJ.

### Luciferase Assay

The *Gpx4* promoter reporter plasmid was constructed in pGL3‐luc plasmid by inserting a PCR‐amplified mouse *Gpx4* promoter fragment at XhoI and HindIII sites using forward primer 5’‐CCGCTCGAGCTCAGGAGGGTGAAGTAGGG and reverse primer 5’‐CCCAAGCTTCAGCCAATGGGAAGCCTG (restriction sites underlined). The construct was sequence‐verified. HEK293 cells were co‐transfected with the *Gpx4* promoter reporter plasmid (*Gpx4*p‐luc) and a renilla luciferase plasmid as an internal control. After 48 h of treatment with MC‐LR, SGI‐1027, or HLM006474, luciferase activity was measured using a dual luciferase assay kit (DL101‐01, Vazyme). *Gpx4* promoter activity was normalized to renilla luciferase and expressed as fold change.^[^ ^15^ ^]^

### C11‐BODIPY Staining

C11‐BODIPY (D3861, Thermo Fisher, USA), a fatty acid analog, fluorescence red (595 nm) in its reduced state and shifts to green (510 nm) upon oxidation.^[^ ^60^ ^]^ HK2 cells seeded in 6‐well plates under various treatments were incubated with C11‐BODIPY dye (10 µm dissolved in DMSO) for 1 h. After washing with PBS, cells were imaged using an Olympus FV3000 confocal fluorescent microscope. Oxidized‐ (O‐BODIPY) and non‐oxidized‐ (N‐BODIPY) forms of were detected at excitation/emission wavelengths of 488/510 and 581/591 nm, respectively. Fluorescence intensity was averaged across 5 randomly‐selected fields, adjusted for cell number, and analyzed using ImageJ.

### Statistical Analysis

Statistical analysis was performed using GraphPad Prism 9. Data normality and variance homogeneity were assessed with Shapiro‐Wilk test and Levene's test, respectively. For comparisons, Student's t‐test (for two groups), one‐way analysis of variance (ANOVA), two‐way ANOVA, or three‐way ANOVA (multiple groups) were applied as appropriate. Results are presented as mean ± SD, or as box‐and‐whisker plots, where the midline represents median, box indicates the 25th–75th percentiles and whiskers represent the minimum and maximum values. ^*^ *P* < 0.05 was considered statistically significant.

## Conflict of Interest

The authors declare no conflict of interest.

## Author Contributions

S.Z. performed methodology, validation, data curation, wrote the original draft, wrote reviewed and edited the final manuscript. Q.G. performed formal analysis, software, data curation, wrote the original draft. Y.P. and H.Z. performed investigation, data curation, wrote the original draft. Q.S. and M.G. performed formal analysis, software, wrote the original draft. Y.G. and L.C. performed validation, wrote reviewed and edited the final manuscript. W.W. performed project administration, wrote reviewed and edited the final manuscript. Y.W. performed methodology, wrote reviewed and edited the final manuscript. W.C. performed conceptualization, resources, acquired funding acquisition, wrote reviewed and edited the final manuscript. Y.W. performed conceptualization, visualization, acquired funding acquisition, wrote reviewed and edited the final manuscript. L.W. performed supervision, project administration, resources, acquired funding acquisition, wrote reviewed and edited the final manuscript.

## Supporting information



Supporting Information

## Data Availability

The data that support the findings of this study are available from the corresponding author upon reasonable request.
