# Supplementary material for: Microcystin‐LR Triggers Renal Tubular Ferroptosis Through Epigenetic Repression of GPX4: Implications for Environmental Nephrotoxicity
Source: Adv Sci (Weinh). 2025 Nov 30;13(9):e14349. doi: 10.1002/advs.202514349 (PMC12903976; doi:10.1002/advs.202514349)
Supplement: Supplementary file 1 — Supporting Information [file ADVS-13-e14349-s001.docx]

**Supplementary information**

**Table S1. The antibodies** **used in this study.**

| Antibodies | Source | Identifier | Working dilution |
| --- | --- | --- | --- |
| MC-LR  GPX4  4-HNE  E-cadherin  α-SMA  Col1α  DNMT1  DNMT3a  DNMT3b  E2F4  NCoR  SMRT  GAPDH  Goat anti-rabbit IgG  Goat anti-mouse IgG | Enzo Life Science  ABclonal  Abcam  Huabio  Huabio  Bioworld  Invitrogen  Cell Signaling Technology  Beyotime  Proteintech  Invitrogen  Invitrogen  Proteintech  FDbio  FDbio | ALX-804-320  A25009  ab48506  EM0502  ET1607-53  BS1530  MA5-16169  #49768  AF1384  10923-1-AP  PA1-844A  PA1-843  60004-1-Ig  FDR007  FDM007 | WB 1:1000; IHC 1:400  WB 1:1000; IHC 1:600  WB 1:1000; IHC 1:200  WB 1:1000  WB 1:5000  WB 1:1000  WB 1:1000; IP 1:100  WB 1:1000; IP 1:50  WB 1:1000  WB 1:1000; ChIP 1:50  WB 1:1000; ChIP 1:100  WB 1:1000; ChIP 1:100  WB 1:100000  WB 1:40000  WB 1:40000 |

**Table S2. The primer sequences used in this study.**

| Target gene | Primer sequence (5’-3’)  Forward Reverse | |
| --- | --- | --- |
| PCR primer  *Gpx4*  *Gapdh*  MSP primer  M-*Gpx4*  U-*Gpx4*  Inp-*Gpx4*  BSP primer  B-*Gpx4*  ChIP primer  C-*Gpx4* | CCCATTCCTGAACCTTTCAA  TATGTCGTGGAGTCTACTGGTGT  TTTTTTAAGGGGATGATTTTGATAC  TTTTAAGGGGATGATTTTGATATGT  CTCTTTAAGGGGATGACTTTGACAC  GTTTTTTAAGGGGATGATTTTGATA  GGGGATGACTTTGACACGC | GCACACGAAACCCCTGTACT  GTCATCATACTTGGCAGGTTTCT  ATACCCAATAATAAAAACGCGAA  CATACCCAATAATAAAAACACAAA  ATGCCCAGTGATAGGGACGCGGG  CCCTACAACCAATAAAAAACCTAAATA  GCCTGAATGAAGGGACGG |
